# Identification of Therapeutic Targets and Prognostic Biomarkers Among Integrin Subunits in the Skin Cutaneous Melanoma Microenvironment

**DOI:** 10.3389/fonc.2021.751875

**Published:** 2021-09-30

**Authors:** Yeltai Nurzat, Weijie Su, Peiru Min, Ke Li, Heng Xu, Yixin Zhang

**Affiliations:** Department of Plastic and Reconstructive Surgery, Shanghai Ninth People’s Hospital, Shanghai JiaoTong University School of Medicine, Shanghai, China

**Keywords:** biomarkers, integrin subunit family, melanoma, skin cutaneous melanoma, cancer genome atlas

## Abstract

The roles of different integrin alpha/beta (ITGA/ITGB) subunits in skin cutaneous melanoma (SKCM) and their underlying mechanisms of action remain unclear. Oncomine, UALCAN, GEPIA, STRING, GeneMANIA, cBioPortal, TIMER, TRRUST, and Webgestalt analysis tools were used. The expression levels of ITGA3, ITGA4, ITGA6, ITGA10, ITGB1, ITGB2, ITGB3, ITGB4, and ITGB7 were significantly increased in SKCM tissues. The expression levels of ITGA1, ITGA4, ITGA5, ITGA8, ITGA9, ITGA10, ITGB1, ITGB2, ITGB3, ITGB5, ITGB6 and ITGB7 were closely associated with SKCM metastasis. The expression levels of ITGA1, ITGA4, ITGB1, ITGB2, ITGB6, and ITGB7 were closely associated with the pathological stage of SKCM. The expression levels of ITGA6 and ITGB7 were closely associated with disease-free survival time in SKCM, and the expression levels of ITGA6, ITGA10, ITGB2, ITGB3, ITGB6, ITGB7, and ITGB8 were markedly associated with overall survival in SKCM. We also found significant correlations between the expression of integrin subunits and the infiltration of six types of immune cells (B cells, CD8+ T cells, CD4+T cells, macrophages, neutrophils, and dendritic cells). Finally, Gene Ontology (GO) enrichment analysis and Kyoto Encyclopedia of Genes and Genomes (KEGG) pathway analysis were performed, and protein-protein interaction (PPI) networks were constructed. We have identified abnormally-expressed genes and gene regulatory networks associated with SKCM, improving understanding of the underlying pathogenesis of SKCM.

## Introduction

Skin cutaneous melanoma (SKCM) is one of the most aggressive and lethal skin cancers (1). In the past decade, incidence of SKCM has increased rapidly worldwide (1). Hence, SKCM poses a major global threat to human health. Recently, associations between molecular biological biomarkers and tumor prognosis during the development of SKCM have elicited great interest. However, our understanding of the etiology and pathogenesis of SKCM could be improved, and more effective prognostic biomarkers are required.

Integrins are glycosylated heterodimers composed of non-covalently bound α and β subunits (2). Specific integrin subunits are known to be closely associated with various tumors, including gallbladder cancer and breast cancer, etc. (3–5). The diversity of integrin function in tumors may be related to differences in the integrin domains. Therefore, we speculate that different integrin subunits may play a role in several biological processes underlying SKCM.

Several comprehensive scientific reviews of the molecular factors underlying SKCM biology, SKCM drug target mechanisms (6), and SKCM prognosis have been published. However, studies clarifying the role of integrins in SKCM are relatively scarce. In the present study, we evaluate the utility of abnormally-expressed integrin subunits as biomarkers in SKCM. In addition, we employ bioinformatics tools for analyzing the underlying mechanisms by which different integrin subunits affect SKCM. Our overall aim is to identify potential biological targets in the integrin subunit family which can be used as biomarkers in SKCM.

## Materials and Methods

### ONCOMINE

The ONCOMINE Database is a multi-functional website based on the Cancer Genome Atlas (TCGA) tumor database (7). Here, we used ONCOMINE to analyze the differentially expressed integrins subunits using a threshold limited by P value<0.05, Fold change ≥2. The specific referencing steps are as follows: the filter of Analysis Type: Cancer *vs.* Normal Analysis; the filter of Cancer Type: Cutaneous Melanoma; the filter of Data Type: mRNA. The data was order by: over-expression: Gene Rank.

### UALCAN

UALCAN (http://ualcan.path.uab.edu/index.html) is an integrated data-mining platform facilitating comprehensive analyses of the cancer transcriptome (8). The functionalities of UALCAN including identifying biomarkers, analyzing expression profile, analyzing gene correlation, and survival analysis (9). In this study, UALCAN database was used to analyze the expression of target genes in primary SKCM, metastasis SKCM and normal samples. The sample information of UALCAN was derived from the TCGA database, and it used the TCGA-Assembler (10) to download the RNA-Seq data of 31 tumors in the TCGA database. RNA-Seq data were obtained for ‘Primary Solid Tumor’ and ‘Solid Tissue Normal’ for each cancer, and the tumor staging in UALCAN was based on the pathological tumor staging data of the American Joint Committee on Cancer (AJCC), which divided the samples into different stages (8). Data visualization was conducted by Highcharts (Highsoft AS Highcharts, http://www.highcharts.com/), a JavaScript library from Highsoft AS (8).

### GEPIA

Developed by Gepia Zefang Tang, is a website that analyzes RNA sequence expression databases based on the TCGA and GTEX projects. Using a deconvolution strategy, it can present cell type information combined with clinical data to help us explore the relationship between cell proportion and prognosis (11). The cancer data of GEPIA was collected from TCGA or GTEx, and the tumor staging definition in GEPIA was based on the pathological tumor staging data of the American Joint Committee on Cancer (AJCC) (12, 13). For the calculation methods of survival analysis, the python package lifeline (https://github.com/CamDavidsonPilon/lifelines) was used for the survival analysis (11). Here, we mainly used the Multiple Gene Analysis module for analyzing the expression of integrins in SKCM at different pathological stages, and correlations between integrin subunit expression and overall survival and disease-free survival.

### STRING

String is a website that can analyze the interaction relationship between genes. In order to understand the interaction relationship between integrin genes in this study, we used STRING website to build a PPI network. Each gene is represented by nodes in the network, and the strength and connections in the network are represented by the color and thickness of the lines.

### GeneMANIA

GeneMANIA is a website for reprocessing PPI network maps (14). GeneMANIA can perform cluster analysis on nodes in the PPI network graph by collecting hundreds of data sets from GEO, Biogrid, Pathway Commons and I2D, so as to provide biological function analysis of each gene in the PPI network graph (14).

### CBioPortal

CBioPortal (http://www.cbioportal.org) is a website used to analyze and visualize cancer genomics data (15). Cbioportal collected the data of 126 tumor genomic studies from TCGA data, based on which cBioPortal can detect the genetic variation, gene network and co-expression of target genes in SKCM (9). Here, we analyzed the gene variation of integrins in SKCM using cBioPortal.

### TIMER

TIMER is a website that analyzes the relationship between tumor purity and immune cell infiltration. Specifically, TIMER can explore the correlation between the degree of infiltration of immune cells in tumor microenvironment and clinical results, somatic mutations, gene expression and somatic copy number changes (16). By collecting the gene expression information of different tumors in the TCGA database, TIMER inferred the abundance of tumor-infiltrated immune cells in the gene expression profile using deconvolution method (17). In this study, the relationship between immune cell infiltration and integrin subunit in SKCM was investigated by using the “gene” module in TIMER.

### Trrust

*TRRUST* (https://www.grnpedia.org/trrust/) is a curated database of human and mouse transcriptional regulatory networks. TRRUST is useful as a tool in predicting these transcriptional regulatory networks (18). Here, TRRUST was used to identify the relationships between selected integrin subunit genes and other target genes.

### Webgestalt

WebGestalt is a website for gene enrichment analysis based on David database. Through the use of WebGestalt, we can quickly visualize the results of the enrichment analysis of DAVID, and at the same time, we can also visualize the results of KEGG pathway (19, 20).

## Results

### 1. Aberrant Expression of Integrin Subunits in SKCM Patients

**Figure 1:**
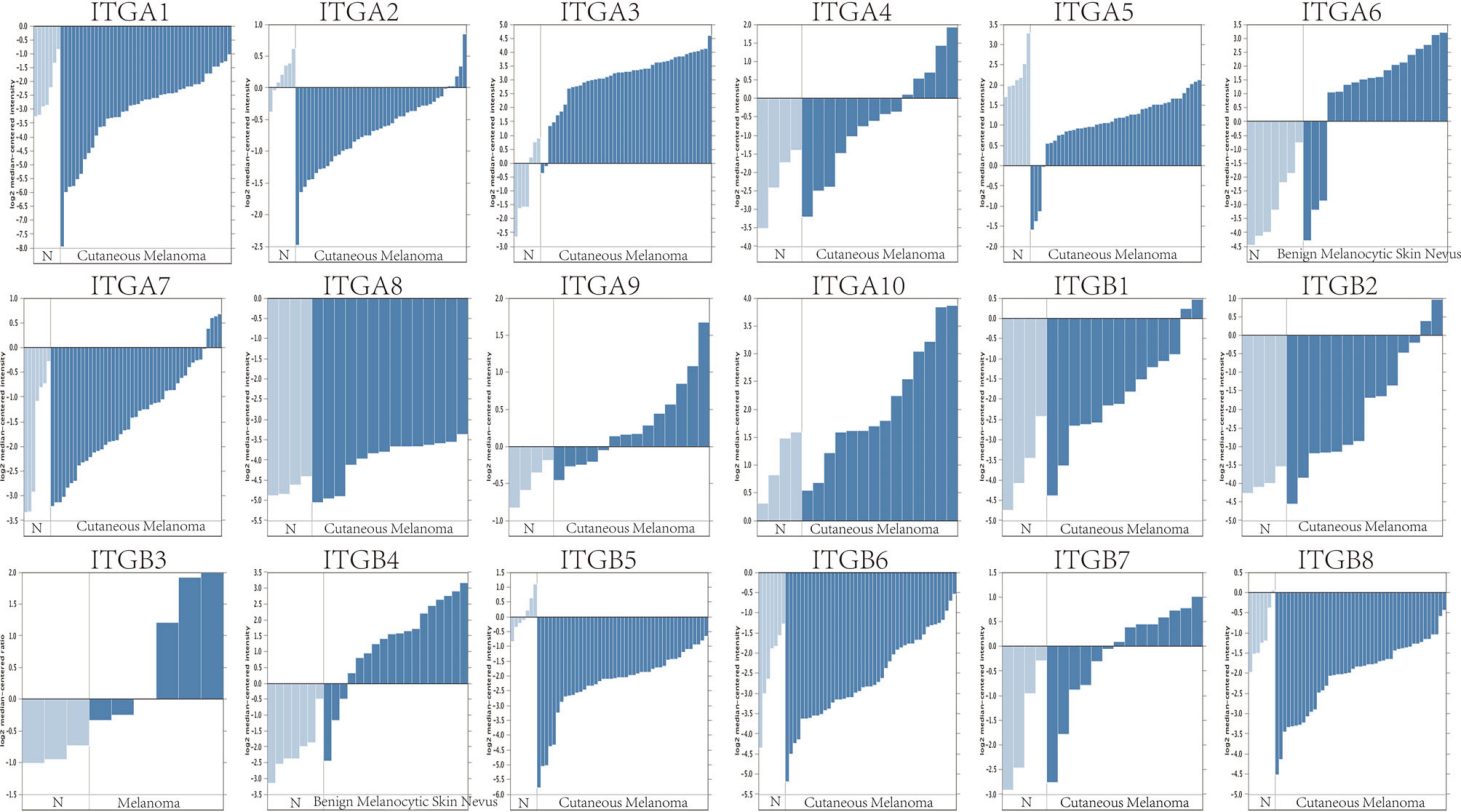
The mRNA expression levels of different integrin subunits in cutaneous melanoma/melanoma/benign melanocytic skin nevus and normal skin tissue (N). The t-test statistic provided in ONCOMINE reflects the magnitude of the difference between the groups. Each bar represents the gene expression of one sample. The p value was set at 0.05.

Firstly, we used the ONCOMINE database to compare the expression profiles of integrin subunits in cutaneous melanoma patients and control groups (**Figure 1** and **Table 1**). The results reveal that the transcription levels of ITGA3, ITGA4, ITGA6, ITGA10, ITGB1, ITGB2, ITGB3, ITGB4, and ITGB7 in Cutaneous Melanoma samples were significantly increased (compared with normal control samples). These results were compiled from several sources. According to Riker et al., the transcription levels of ITGA4, ITGA10, ITGB1, ITGB2, and ITGB7 in SKCM patients were significantly increased (compared with normal skin tissue), with fold changes of 3.224, 2.074, 3.525, 3.968, and 2.84 respectively (21). Haqq et al. reported that the transcription level of ITGB3 in melanoma patients was significantly increased (compared with normal skin tissue), with a fold change of 3.147 (22). Talatov et al. reported that the transcription levels of ITGA3, ITGA6, and ITGB4 in cutaneous melanoma were significantly increased (compared with normal tissue), with fold changes of 14.807, 16.226, and 10.644 (23). The significant differences in the expression levels of other integrin subunits were not observed between SKCM patients and normal patients. For specific sample information, please refer to the references mentioned in **Table 1**.

**Table 1:** Aberrant expression of integrin subunits in SKCM patients.

GENE	CANCER TYPE	FOLD CHANGE	p-value	t-test	Reference	PMID
ITGA3	Cutaneous Melanoma *vs.* Normal	14.807	9.58E-05	7.105	Talantov Melanoma	PMID: 16243793
ITGA4	Cutaneous Melanoma *vs.* Normal	3.224	0.013	2.75	Riker Melanoma	PMID: 18442402
ITGA6	Benign Melanocytic Skin Nevus *vs.* Normal	16.226	1.71E-05	5.491	Talantov Melanoma	PMID: 16243793
ITGA10	Cutaneous Melanoma *vs.* Normal	2.074	0.015	2.55	Riker Melanoma	PMID: 18442402
ITGB1	Cutaneous Melanoma *vs.* Normal	3.525	0.011	2.983	Riker Melanoma	PMID: 18442402
ITGB2	Cutaneous Melanoma *vs.* Normal	3.968	3.61E-04	4.213	Riker Melanoma	PMID: 18442402
ITGB3	Melanoma *vs.* Normal	3.147	0.006	3.681	Haqq Melanoma	PMID: 15833814
ITGB4	Benign Melanocytic Skin Nevus *vs.* Normal	10.644	2.76E-03	7.278	Talantov Melanoma	PMID: 16243793
ITGB7	Cutaneous Melanoma *vs.* Normal	2.84	0.043	2.21	Riker Melanoma	PMID: 18442402

**Figure 2:**
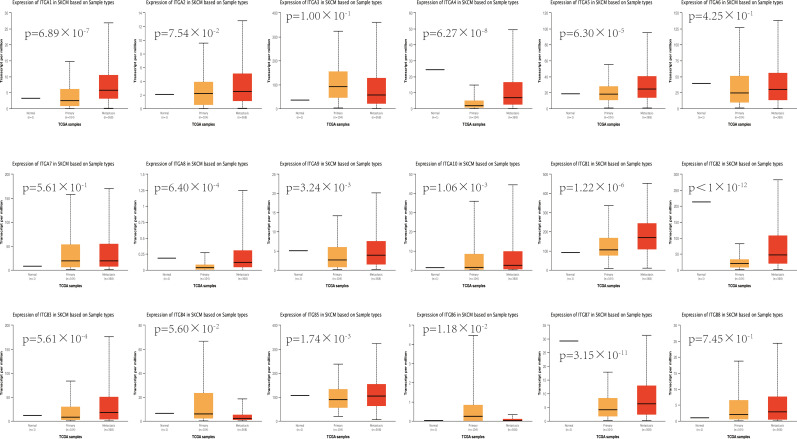
The expression levels of different integrin subunits in primary SKCM samples and metastatic SKCM samples (UALCAN). Student’s t test was used to generate a p value. The p value was set at 0.05.

To further validate the above results, we assessed the expression levels of different integrin subunits in primary SKCM samples and metastatic SKCM samples with UALCAN. Our analysis revealed that the transcriptional levels of ITGA1 (p = 6.89×10^-7^), ITGA4 (p = 6.27×10^-8^), ITGA5 (p = 6.30×10^-5^), ITGA8 (p = 6.40×10^-4^), ITGA9 (p = 3.24×10^-3^), ITGA10 (p =1.06×10^-3^), ITGB1 (p = 1.22×10^-6^), ITGB2 (p < 1×10^-12^), ITGB3 (p = 5.61×10^-4^), ITGB5 (p = 1.74×10^-3^), and ITGB7 (p = 3.15×10^-11^) in metastatic SKCM tissues were significantly increased compared with primary SKCM tissues (**Figure 2**). Whereas, the expression level of ITGB6 (p = 1.18×10^-2^) in metastatic SKCM tissue was decreased compared with primary SKCM tissue (**Figure 2**). All other comparisons do not meet our threshold by p value<0.05 and fold change ≥ 2.

**Figure 3:**
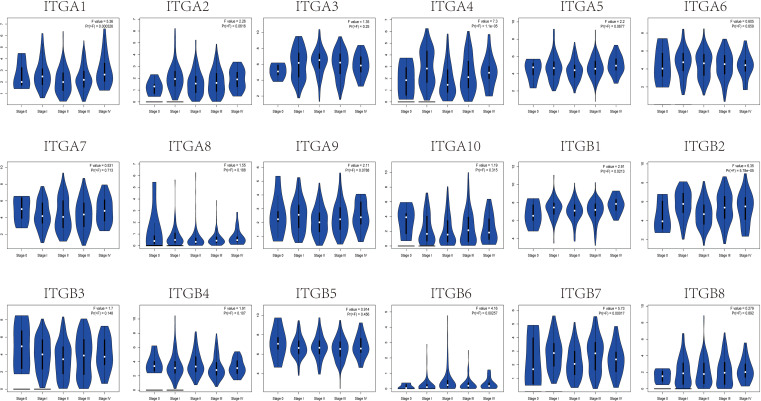
Correlation between different expressed integrin subunits and the pathological stage of SKCM patients (GEPIA). A P value less than 0.05 was used to determine statistical difference. Student’s t test was used to generate a p value for expression or pathological stage analysis.

**Figure 4:**
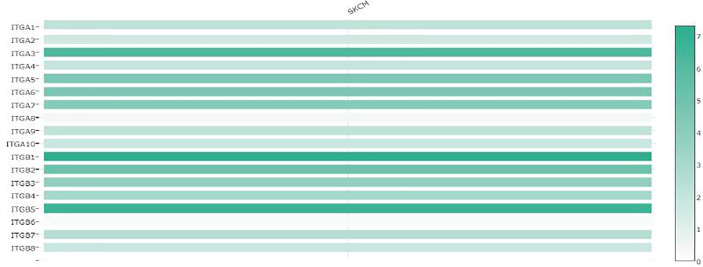
The relative expression levels of integrin subunits in SKCM.

Lastly, to clarify the clinical significance of the observed changes in integrin subunit expression, we analyzed the correlations between integrin subunit expression and the pathological stage of SKCM using GEPIA. The analysis revealed that ITGA1 (p = 0.000326), ITGA4 (p = 1.1×10^-5^), ITGB1 (p = 0.0213), ITGB2 (p = 5.78×10^-5^), ITGB6 (p = 0.00257), and ITGB7 (p = 0.00017) were notably associated with the pathological stage of SKCM (**Figure 3**). To clarify the gene expression rank of integrin subunit gene expression, we analyzed the expression levels of these genes in SKCM tissue using the GEPIA website (**Figure 4**). The results reveal that the expression level of ITGB1 in SKCM patients was highest among the integrin genes of interest.

### 2. The Prognostic Value of Integrin Subunits in SKCM Patients

**Figure 5:**
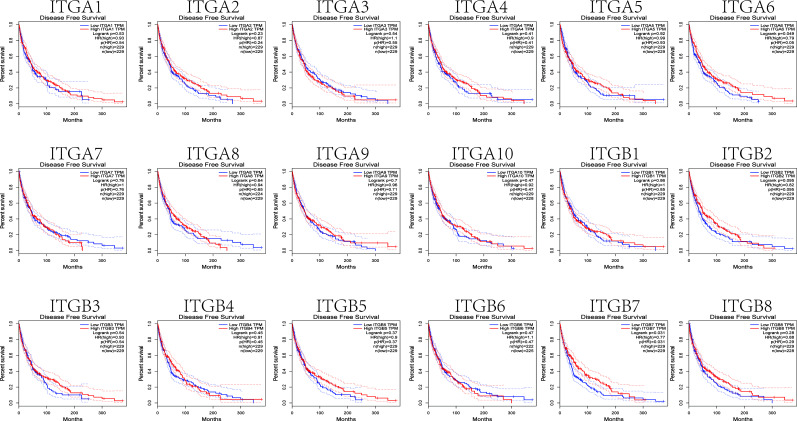
The prognostic value of differentially-expressed integrin subunits in the disease-free survival curve (GEPIA) for SKCM patients. Prognostic analysis was performed using a Kaplan–Meier curve. The p value was set at 0.05.

**Figure 6:**
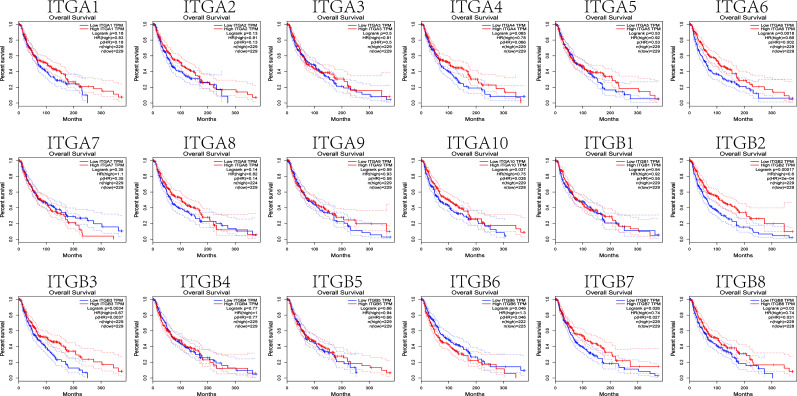
The prognostic value of differentially-expressed integrin subunits in the overall survival curve (GEPIA) for SKCM patients. Prognostic analysis was performed using a Kaplan–Meier curve. The p value was set at 0.05.

We next investigated correlations between integrin subunit expression levels and patient prognosis using GEPIA. The results of this analysis reveal that integrin subunit expression and disease-free survival time in SKCM patients are related. Two of these correlations — those involving ITGA6 and ITGB7 — were statistically significant (**Figure 5**). We next analyzed the associations between integrin subunit expression levels and overall survival in SKCM patients. The results reveal that the expression levels of ITGA6, ITGA10, ITGB2, ITGB3, ITGB6, ITGB7, and ITGB8 were remarkably correlated with overall survival in SKCM patients (**Figure 6**).

### 3. Relationship Between Integrin Subunit Expression and Immune Cell Infiltration

**Figure 7:**
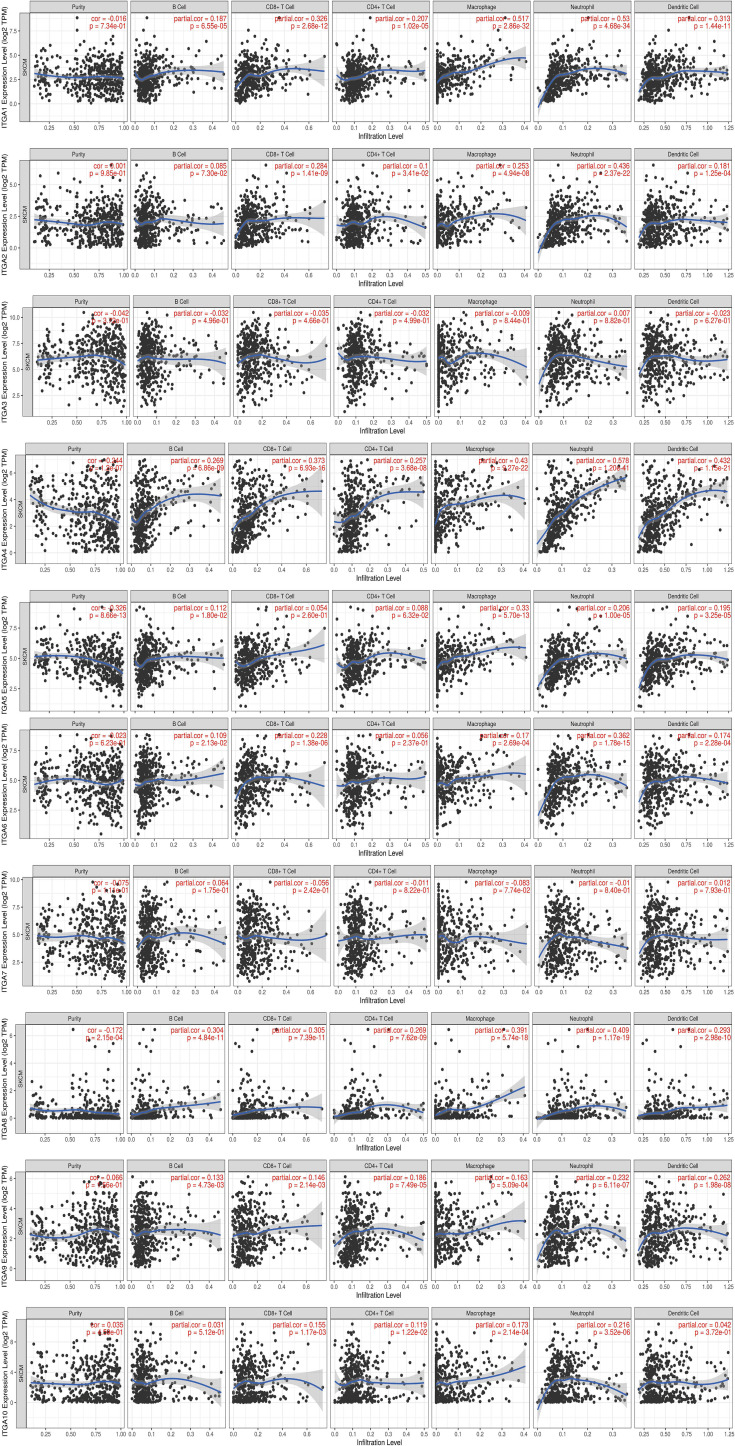
Immune infiltration associated with ITGA1 – ITGA10 in SKCM patients (TIMER). Spearman correlation coefficient was used for statistical analysis. A P value less than 0.05 was used to determine statistical difference.

**Figure 8:**
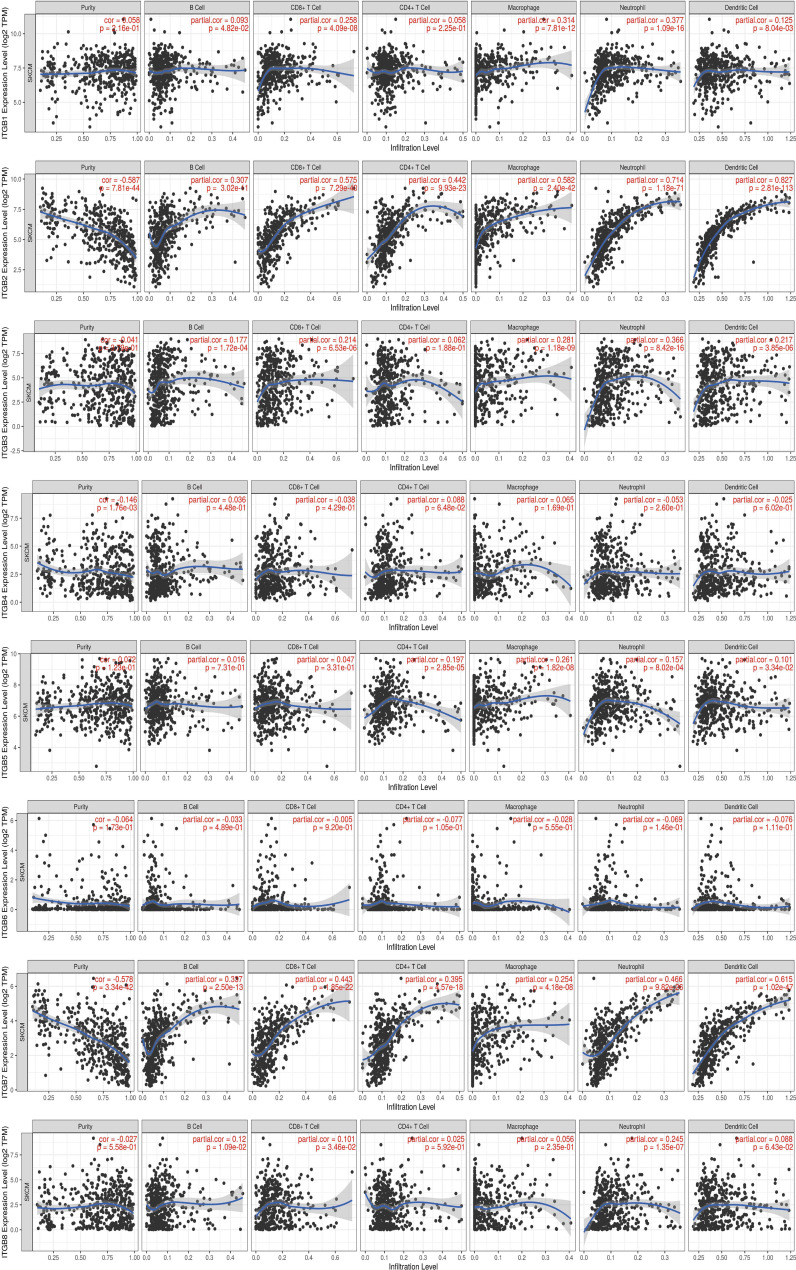
Immune infiltration associated with ITGB1 – ITGB8 in SKCM patients (TIMER). Spearman correlation coefficient was used for statistical analysis. A P value less than 0.05 was used to determine statistical difference.

Tumor immune microenvironment (TIME) refers to the mutual environment composed of tumor cells and their surrounding immune cells. Evidence is accumulating to suggest that immune cell infiltration is closely related with cancer occurrence and resistance, and the degree of immune cell infiltration can evaluate the effectiveness of clinical tumor immunotherapy (16, 24). If the expression of the gene in SKCM TIME is related to the infiltration of immune cells and tumor purity, it is suggested that the gene may become a target of tumor immunotherapy. To further explore the function of integrin subunits in SKCM, the immune effects of integrin subunits were analyzed using the TIMER website (**Figures 7** and **8**).

The expression of *ITGA4, ITGA8, ITGB2*, *ITGB7* and *ITGB8* was correlated with the infiltration of all types immune cell and tumor purity in the SKCM TIME. *ITGA5* was related with tumor purity, B cell, Macrophage cell, Neutrophil cell, and Dendritic cell invasion in SKCM TIME. The expression of *ITGA1* and *ITGA9* was correlated with the infiltration of all types immune cell in the SKCM TIME, but not tumor purity. The expression of *ITGA2* was correlated with the infiltration of CD8+/CD4+ T cell, Macrophage cell, Neutrophil cell and Dendritic cell in SKCM patients. *ITGA6*, *ITGB1* and *ITGB3* was correlated with B cell, CD8+ T cell, Macrophage cell, Neutrophil cell and Dendritic cell infiltration. *ITGA10* was associated with CD4+/CD8+ T cell, Macrophage cell and Neutrophil cell infiltration. *ITGB5* was related with the invasion of CD4+ T cell, Macrophage cell, Neutrophil cell and Dendritic cell. *ITGB8* was correlated with CD8+ T cell, B cell and Neutrophil cell invasion.

### 4. Integrin Subunit Genetic Alteration and Protein Interaction Network in 1SKCM Patients

**Figure 9:**
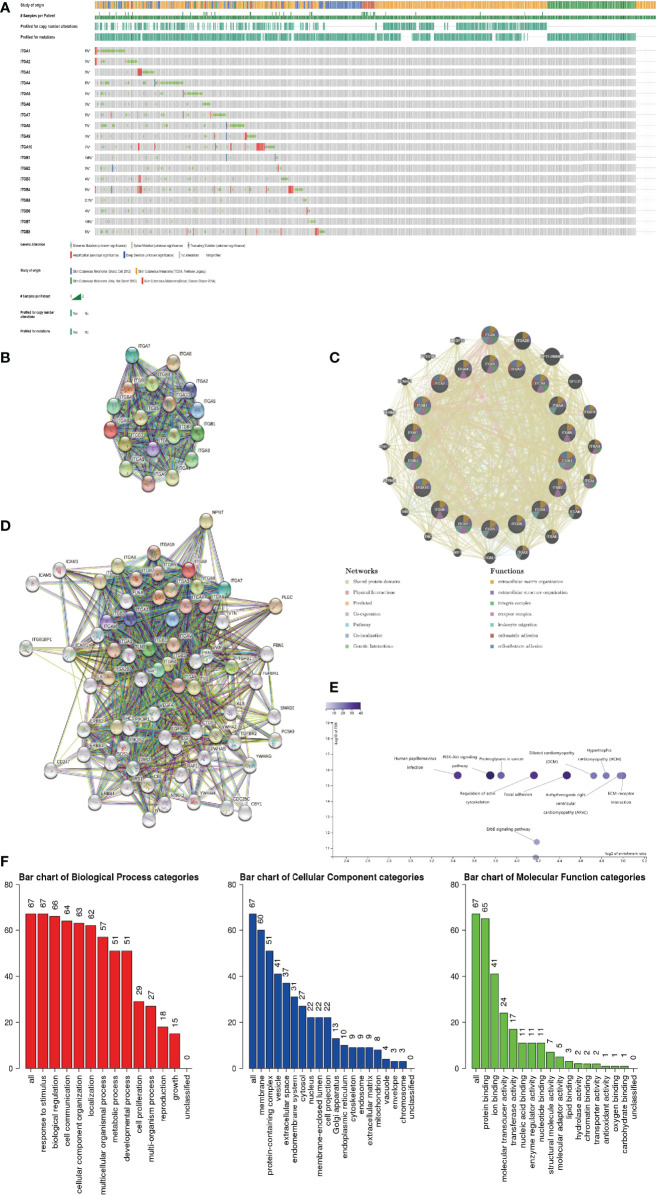
**(A)** Summary of genetic alterations in different integrin subunits in SKCM. **(B, C)** Protein-protein interaction network of different integrin subunits. **(D)** Gene-gene interaction network of different integrin subunits and 48 most frequently altered neighboring genes. **(E, F)** Enrichment analysis of different integrin subunits and 48 most frequently altered neighboring genes in SKCM (Webgestalt). **(E)** Bar plot of KEGG enriched terms. **(F)** Bar plot of GO enrichment in biological process terms, cellular component terms, and molecular function terms.

Firstly, we explored genetic alterations in the integrin subunit family in SKCM using the cBioportal website. The results of this analysis reveal that *ITGA1, ITGA2, ITGA3, ITGA4, ITGA5, ITGA6, ITGA7, ITGA8, ITGA9, ITGA10, ITGB1, ITGB2, ITGB3, ITGB4, ITGB5, ITGB6, ITGB7*, and *ITGB8* were changed in 6%, 3%, 3%, 8%, 5%, 3%, 5%, 7%, 4%, 7%, 1.6%, 3%, 4%, 8%, 2.1%, 4%, 1.9%, and 6% of the queried SKCM samples, respectively (**Figure 9A**). Next a PPI network of the integrin subunit family was constructed to explore possible interactions. A PPI network with 18 nodes and 152 edges was constructed using STRING (**Figure 9B**). According to the statistical results reported by the String website, the P-value of PPI enrichment was <1.0e-16. Further analysis using GeneMANIA revealed that the above-mentioned integrin subunits were mainly involved in extracellular matrix organization, extracellular structure organization, integrin complex, receptor complex, and leukocyte migration (**Figure 9C**). To further corroborate these observations, we used STRING to analyze the top 48 most interacting neighboring genes associated with 18 integrin subunits. The results demonstrate that ALB, CBL, CBY1, CD247, CDC25C, CTGF, EGF, EGFR, ERBB2, ERBB3, ERBB4, FBN1, FLNA, FN1, GRB2, ICAM1, ICAM3, ICAM5, ILK,IRS1, ITGA1, ITGA10, ITGA2, ITGA2B, ITGA3, ITGA4, ITGA5, ITGA6, ITGA7, ITGA8, ITGA9, ITGAL, ITGAM, ITGAV, ITGAX, ITGB1, ITGB1BP1, ITGB2, ITGB3, ITGB4, ITGB5, ITGB6, ITGB7, ITGB8, NPNT, NTRK1, NTRK2, PCSK9, PIK3R1, PIK3R2, PLEC, PXN, RAF1, SHC1, SMAD2, SOS1, SRC, TGFB1, TGFBR1, TGFBR2, TLN1, VEGFA, VTN, VWF, YWHAB, YWHAG, YWHAH, and YWHAZ are all involved in either the regulation or function of integrin subunit family members in SKCM patients (**Figure 9D**).

### 5. Gene Ontology and KEGG Pathway Analysis of Integrin Subunits in SKCM Patients

Go and KEGG pathway analysis for 18 integrin subunits and top 48 most interacting neighboring genes was performed using the Webgestalt website (**Figures 9E, F**). In the KEGG pathway analysis, the top 5 pathways identified were ECM-receptor interaction, arrhythmogenic right ventricular cardiomyopathy, hypertrophic cardiomyopathy, dilated cardiomyopathy, and focal adhesion. In the GO enrichment analysis, the results pertaining to biological processes were response to stimulus, biological regulation, and cell communication. For the results pertaining to the analysis of cellular components, the top five cellular components were membrane, protein-containing complex, vesicle, extracellular space, and endomembrane system. For the results pertaining to the analysis of molecular function, the top 5 molecular functions were protein binding, ion binding, molecular transducer activity, transferase activity, and nucleic acid binding.

## Discussion

Recent studies indicate that ITGA1 is associated with melanoma proliferation *via* the regulation of miR-3065-5p (25). In addition, as a mediator of cell-cell and cell-matrix adhesion, ITGA1 was differentially expressed in melanoma samples (26). In the present study, we found that ITGA1 expression levels in SKCM tissues are correlated with different stages of SKCM and with migration in SKCM. ITGA1 protein mediate the adhesion of extracellular matrix proteins (27). We hypothesized that ITGA1 protein in SKCM promote the adhesion of melanocytes to the epidermal basement membrane (28), thus affecting the metastasis of SKCM. Besides, we found a positive correlation between the expression of *ITGA1* and infiltration of all types of immune cell, but not tumor purity (**Figure 7**). Therefore, the effect of ITGA1 in tumor immune therapy may need further verification.

ITGA2 plays a role in melanoma by regulating the expression GTSE1, thereby restoring the epithelial-to-mesenchymal transition in SKCM (29). However, according to our results, the increased expression of ITGA2 may only be related to the infiltration of some immune cells, and this infiltration of immune cells does not lead to the reduction of tumor purity in SKCM TIME, suggesting that the infiltration of immune cells may not play a decisive role in SKCM.

Schumacher et al. reported that flocculating material observed around melanoma cells or nests contains basal membrane protein components, particularly ITGA3 (30). In line with previous research, we found that ITGA3 expression in SKCM tissues was significantly increased (in comparison with non-tumor tissue). Moreover, it is worth mentioning that ITGA3 has most biological functions compared with other integrin subunits, suggesting that it may play a central role in integrin subunits gene clusters (**Figure 9C**).

ITGA4 is closely related to the occurrence and development of melanoma, and ITGA4 can promote the metastasis of melanoma by promoting the aggregation of melanoma cells in the lymphatic system (31). J. Zhao et al. reported that ITGA4 down-regulation inhibits the adhesion and migration of melanoma cells *in vitro* and *in vivo* (32). The immunomodulatory mechanism of ITGA4 in melanoma has also been reported in previous studies. Geherin et al. reported that IL-10+ B1 cells are part of the skin immune system and require α4β1 integrin for homing into the skin (33). In addition, Kobayashi et al. reported that IL-10+ B1a B cells suppress melanoma tumor immunity by inhibiting Th1 cytokine production in tumor-infiltrating CD8+ T cells (34). According to our results, we speculated that the differentially expressed ITGA4 may recruit immune cells by effecting leukocyte migration, reduce the percentage of melanoma cells in SKCM TIME by affecting cell matrix adhesion in SKCM TIME, and ultimately affect the metastasis and tumor stage of SKCM (**Figures 7** and **9C**).

ITGA5 forms a receptor for extracellular fibronectin, which known to be involved in the formation of malignant tumor cells and tumor vascular systems (35). ITGA5 is known to play a role in melanoma by regulating miR-148b (36). Combined with our results, we hypothesized that ITGA5 may affect the infiltration of immune cells and tumor purity in SKCM TIME by regulating leukocyte migration (**Figures 7** and **9C**).

Specific ITGA6 variants were reported to be associated with a decreased risk of melanoma, and Luo et al. reported that ITGA6 can be considered a prognostic gene for uveal melanoma (37, 38). We also found that abnormally expressed ITGA6 is a potential prognostic biomarker in SKCM, and we speculate that missense mutation is one of the main reasons for the abnormal expression of ITGA6 (**Figure 9A**).

ITGA8 is a transmembrane cell surface receptor belonging to the alpha integrin family (39). To date, several studies have linked ITGA8 and tumorigenesis (40, 41). In addition, anti-ITGA8 therapies have been reported to play a role in the treatment of lupus and other glomerular diseases (42). Tumor cells express antigens that mediate recognition by CD8+ T cells and the infiltration of CD8+ T cells in SKCM TIME is a very important part of immunotherapy (43). Combined with our results, we speculated that interference against ITGA8 expression could effectively increase the infiltration of CD8+ T cells in SKCM TIME and reduce the percentage of tumor cells in SKCM, which is a potential target of SKCM targeted therapy (**Figure 7**).

Several studies have reported a close association between ITGA9 and cell adhesion, proliferation, and migration (44). Changes in ITGA9 expression levels affect the interaction between tumor cells and the extracellular matrix (45). *ITGA9* is a host gene for various long non-coding RNAs, including LncCCAT1 and HOXA11-AS. Therefore, proliferation, apoptosis, metastasis of melanoma cells may be modulated *via* regulation of *ITGA9*-related non-coding RNAs (46, 47). In the present study *ITGA9* was positively correlated with the invasion of several immune cells in SKCM TIME, but not tumor purity (**Figure 7**). Combined with our results and previous reports, we suggest that ITGA9 in SKCM may mediate cell-cell communication between cancer cells and their microenvironment by influencing the formation of integrin and receptor complexes, and ultimately effect SKCM metastasis and progression (**Figure 9C**) (48).

ITGA10 is a transmembrane glycoprotein involved in cell adhesion and integrin-mediated signaling pathways (49). ITGA10 is associated with various cancers development and metastasis, and variants in *ITGA10* are associated with changes of melanoma risks (50). In the present study, we found that ITGA10 expression was up-regulated in SKCM tissues (in comparison with control tissues), and this abnormal expression of ITGA10 may be the results of amplification mutations or the missense mutations (**Figure 9A**).

Previous studies have confirmed the correlation between ITGB1 expression and SKCM metastasis (51). In line with previous studies, we report aberrant ITGB1 expression in SKCM, and we reveal that ITGB1 expression is closely associated with SKCM metastasis by effecting cell migration (**Figure 2**) (52).

According to a bioanalysis conducted by Jun Zhu et al., ITGB2 was one of the top hub genes in malignant melanoma, and its expression was related to overall survival and disease-free survival (53). Combined with our results, we believe that ITGB2 is one of the integrin subunits most closely related to SKCM. ITGB2 plays a variety of roles in integrin complex, including extracellular matrix formation, Integrin complex formation, leukocyte migration, etc. Through the above functions, the highly expressed ITGB2 can effectively recruit T cells in SKCM TIME and reduce the purity of tumor cells in SKCM, so the overexpression of ITGB2 may effectively improve the effectiveness of immunotherapy against SKCM and improve the overall survival rate.

ITGB3 may play a vital role in the treatment of melanoma. Inhibition of ITGB3-SRC-STAT3 pathway activation can sensitize tumor-repopulating cells to the effects of IFN-α, and enhance the overall efficacy of melanoma treatment (54). In addition, the ADAR1-ITGB3 network may also play a central role in acquisition of an invasive phenotype in metastatic melanoma (55, 56). Transcription of ITGB3 gene induces the expression of NME1, a metastatic suppressor, in melanoma (57). According to our results, we hypothesized that the abnormal expression of ITGB3 in SKCM suggesting its value as a prognostic marker.

ITGB4 and ITGA6 are heterodimeric cambium adhesin receptors. ITGB4 has a long cytoplasmic domain and has unique cytoskeleton and signaling functions (58, 59). In addition, a mutation in ITGB4 has been identified in a metastasis sample taken from acral melanoma patients (60). The findings presented here are consistent with this previous research. In particular, ITGB4 expression was significantly increased in SKCM tissues compared with non-tumor tissues (**Figure 1**).

*ITGB5*, which is located between 13:133161078 and 13:139609422 in the SSC13Q41 region, encodes the integrin β5 subunit, and this coordinates with the αV subunit to produce the integrin αVβ5 (61). Reports concerning the role of ITGB5 in melanoma are scarce.

ITGB6 is extensively involved in wound healing and the pathogenesis of a variety of diseases, including fibrosis and cancer (62). Previous studies have identified abnormal expression of ITGB6 in SK-Mel-28 human melanoma cells (63). In line with previous findings, we demonstrate here that ITGB6 expression was positively associated with SKCM tumor stage (**Figure 3**).

There are only a few reports concerning ITGB7 expression in melanoma and its potential role. However, we found that ITGB7 was not only abnormally expressed in SKCM, but also correlated with the prognosis of SKCM. The expression of ITGB7 in SKCM TIME was positively correlated with immune cell infiltration and negatively correlated with tumor purity. integrin subunits target therapy such as etrolizumab may play a role in the treatment of SKCM by mediated the infiltration of immune cells, of course, this needs further trial verification.

As for ITGA7 and ITGB8, there have been few reports confirming their connection to SKCM, and we did not find meaningful results in our study, we prefer to leave this question open.

Integrins form a heterodimer with α subunit and β subunit, and there is an intimate connection between integrin α subunits and β subunits (**Figure 9B**). Considering the biological functions of integrin subunits family and the results of our gene enrichment analysis (**Figures 9E, F**), we believe that integrin subunits family, which are mainly distributed in cell membrane and protein-containing complex, may affect protein-binding in SKCM by participating in biological processes such as cell communication, cellular component organization and response to stimulus and other biological processes. Further, these biological functions of the integrin family may also be inseparable from 48 genes (**Figure 9D**) that interact with them.

## Conclusion

In conclusion, ITGA4, ITGB2 and ITGB7 was identified as novel biomarkers which may assist in the design of new immunotherapeutic drugs and server as diagnostic biomarkers. However, there are several limitations with our research. Firstly, *in vivo* and *in vitro* research should be conducted to verify our results. Secondly, this research was conducted mainly based on public databases. As we have not explained all the statistics and code information used in these databases in detail, this may cause some confusion in non-specialist readers.

## Data Availability Statement

The datasets presented in this study can be found in online repositories. The names of the repository/repositories and accession number(s) can be found in the article/supplementary material.

## Author Contributions

YN and WS carried out the experiment and wrote the manuscript with support from PM and KL, HX, and YZ helped supervise the project and conceived the original idea. All authors contributed to the article and approved the submitted version.

## Conflict of Interest

The authors declare that the research was conducted in the absence of any commercial or financial relationships that could be construed as a potential conflict of interest.

## Publisher’s Note

All claims expressed in this article are solely those of the authors and do not necessarily represent those of their affiliated organizations, or those of the publisher, the editors and the reviewers. Any product that may be evaluated in this article, or claim that may be made by its manufacturer, is not guaranteed or endorsed by the publisher.

## Abbreviations

SKCM, skin cutaneous melanoma; AJCC, American Joint Committee on Cancer.
